# A Geographical Information System Based Approach for Integrated Strategies of Tick Surveillance and Control in the Peri-Urban Natural Reserve of Monte Pellegrino (Palermo, Southern Italy)

**DOI:** 10.3390/ijerph15030404

**Published:** 2018-02-27

**Authors:** Alessandra Torina, Valeria Blanda, Marcellocalogero Blanda, Michelangelo Auteri, Francesco La Russa, Salvatore Scimeca, Rosalia D’Agostino, Rosaria Disclafani, Sara Villari, Vittoria Currò, Santo Caracappa

**Affiliations:** Istituto Zooprofilattico Sperimentale della Sicilia “A.Mirri”, Via G. Marinuzzi 3, 90100 Palermo, Italy; alessandra.torina@izssicilia.it (A.T.); valeria.blanda@gmail.com (V.B.); marcelloblanda@hotmail.com (M.B.); larussafrancesco81@gmail.com (F.L.R.); salvatore.scimeca70@gmail.com (S.S.); rosalia.dagostino1@gmail.com (R.D.); rosariadisclafani@gmail.com (R.D.); sara.villari@izssicilia.it (S.V.); vittoria.curro@izssicilia.it (V.C.); santo.caracappa@izssicilia.it (S.C.)

**Keywords:** Ixodidae ticks, GIS, ecological analysis, urban park, Sicily

## Abstract

Ticks (Acari: Ixodidae) are bloodsucking arthropods involved in pathogen transmission in animals and humans. Tick activity depends on various ecological factors such as vegetation, hosts, and temperature. The aim of this study was to analyse the spatial/temporal distribution of ticks in six sites within a peri-urban area of Palermo (Natural Reserve of Monte Pellegrino) and correlate it with field data using Geographical Information System (GIS) data. A total of 3092 ticks were gathered via dragging method from June 2012 to May 2014. The species collected were: *Ixodes ventalloi* (46.09%), *Hyalomma lusitanicum* (19.99%), *Rhipicephalus sanguineus* (17.34%), *Rhipicephalus pusillus* (16.11%), *Haemaphisalis sulcata* (0.36%), *Dermacentor marginatus* (0.10%), and *Rhipicephalus turanicus* (0.03%). GIS analysis revealed environmental characteristics of each site, and abundance of each tick species was analysed in relation to time (monthly trend) and space (site-specific abundance). A relevant presence of *I. ventalloi* in site 2 and *H. lusitanicum* in site 5 was observed, suggesting the possible exposure of animals and humans to tick-borne pathogens. Our study shows the importance of surveillance of ticks in peri-urban areas and the useful implementation of GIS analysis in vector ecology; studies on temporal and spatial distribution of ticks correlated to GIS-based ecological analysis represent an integrated strategy for decision support in public health.

## 1. Introduction

Ticks (Acari: Ixodidae) are the most common vectors of infectious animal diseases and they pose a serious threat to humans, pets, wild animals, and livestock worldwide. Ticks are involved in the transmission of several tick borne pathogens (TBPs), such as *Rickettsia*, *Babesia*, *Theileria*, *Borrelia*, and *Coxiella*; some of them can also be agents of zoonosis [1]. Effective vaccines are not yet available for the majority of tick-borne pathogens [2,3,4]. Prevention methods against vectors are, therefore, to date, the most effective tools against tick borne pathogens. Monitoring of tick distribution and identification of the greatest risk environments is one of the most useful strategies to prevent the risk of tick-borne diseases. Tick questing activity, reproduction, and survival depend on several factors, including vegetation coverage, host availability, moisture, and temperature [5,6].

The great ecological and climatic diversity across Italy make this country a very favourable area for tick development and diffusion. In fact, 40 tick species were recorded in Italy and correlated to TBPs, including *Rhipicephalus sanguineus* (Latreille, 1806), *Rhipicephalus bursa* (Canestrini and Fanzago, 1878), *Ixodes ricinus* (Linnaeus, 1758), *Dermacentor marginatus* (Sulzer, 1776), *Hyalomma marginatum* (Koch, 1844), and *Haemaphysalis sulcata* (Canestrini and Fanzago, 1878). Tick-borne diseases (TBDs) are, thus, widespread in Italy, especially rickettsiosis with 10,069 human cases from 1996 to 2010 [7], with Sicily, Sardinia, Latium, and Calabria reported as the most affected regions. Accordingly, most of the zoonotic pathogens detected in ticks collected from humans are *R. conorii conorii* (Brumpt, 1932), *R. monacensis* (Simser et al., 2002) [8], *R. massiliae* (Beati and Raoult, 1993) [9], *R. slovaca* (Sekeyova et al., 1998) [10], *R. helvetica* (Beati et al., 1993) [11], and *R. aeschlimannii* (Beati et al., 1997) [12,13,14], leading to various clinical manifestations of Spotted Fever Group Rickettsioses [15,16]. In addition, pathogens *Anaplasma phagocythophilum* (Foggie, 1949) and *Candidatus* Neoehrlichia mikurensis (Kawahara et al., 2004) [17] were detected in *Ixodes ricinus* in Italy, with a risk for human granulocytic anaplasmosis and *Candidatus* Neoehrlichia mikurensis-related disease [14]. Furthermore, different tick species in Italy are two- or three-host ticks (i.e., *H. marginatum*—*I. ricinus*), potentially affecting various hosts including: (i) farmed, economically-relevant animals such as cattle, sheep, and horses, causing low to severe forms of babesiosis and theileriosis in such species [18]; (ii) wild animals such as wild boars, badgers, and rabbits [19]; (iii) pets such as dogs [20]. Thus, humans represent occasional hosts for various tick species, and the possibility of tick transfer from animals to humans in Italy is relevant in both rural areas (i.e., farmers) and sub-urban and urban areas. Town parks and suburban green zones, constituting areas of urban recreational activity for families, athletes, and pets, represent suitable environments for contact with bloodsucking arthropods, as for example, ticks. Nevertheless, few studies have been conducted in Italy to investigate tick abundance and seasonal dynamics in urban and peri-urban parks used for recreational activities [21,22].

The control of tick vectors for limiting TBDs, especially in urban/peri-urban areas, is still a problematic issue; in fact, although various strategies have been employed, including vaccination against TBPs, biological control, and tick decoys, the extensive use of acaricides and repellents are still recognized as the most incisive control methods [23]. However, in recent years, different studies demonstrated that the use of acaricides lead to the development of acaricide resistance in ticks [24], a risky condition which could cause future failures in control programs. Regarding both acaricides and repellents, another major limit, especially in areas with a high human presence, is the relative toxicity of such mostly synthetic substances that may represent a threat for public health; furthermore, biodegradation of synthetic acaricides/repellents is a major issue both for public health and ecological implications. In such a context, novel approaches are arising, including the research of natural substances with the potential to act as acaricides/repellents (i.e., plant-derived essential oils) [23,25], which should be a preferable option for avoiding toxicity for humans or other non-target animal species, contamination of animal products, and environmental pollution.

Furthermore, infectious and parasitic diseases transmitted by vectors are strongly influenced by environmental determinants, leading to the necessity of an integrated ecological and environmental analysis. In such a context, recently, the Geographic Information System (GIS) had progressively become an effective analysis tool [26].

The application of this tool has increased from very specific areas to ever more broad fields, including human and veterinary medicine, epidemiology, and entomology. GIS application in the health field is taking on an increasingly important role as a support instrument for disease mapping, ecological analysis, and risk assessment. The GIS allows managing, analysing, and correlating the health and/or entomological data with environmental data, such as vegetation, altitude and land cover. This approach is called ecological analysis, and it is a great tool in the identification of environmental risk factors (risk assessment). It has been applied to the study of vectors and bacterial, viral, and parasitic diseases [27,28,29].

This study was aimed at the analysis, through GIS application, of spatial and temporal distribution of free-living ticks in the Natural Reserve of Monte Pellegrino, in Palermo (Italy), a peri-urban area of the city used by families, walkers, and pets for recreational and sportive activities.

## 2. Materials and Methods

### 2.1. Collection Sites

**Figure 1:**
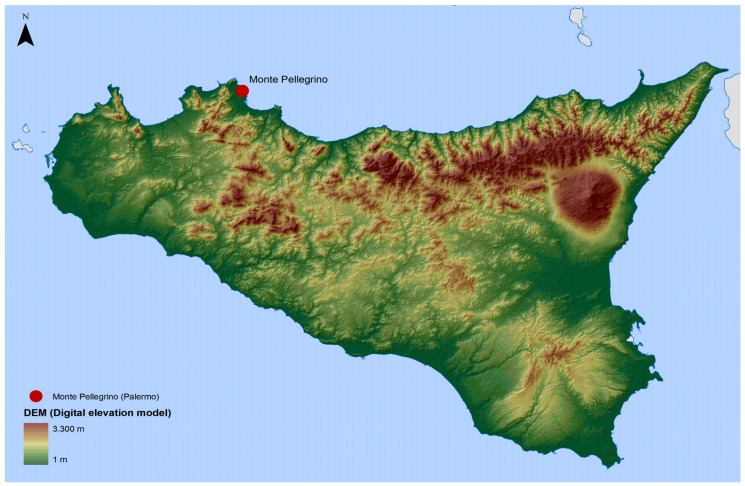
Position of Monte Pellegrino, in the Northern part of Palermo (Sicily, Italy).

**Figure 2:**
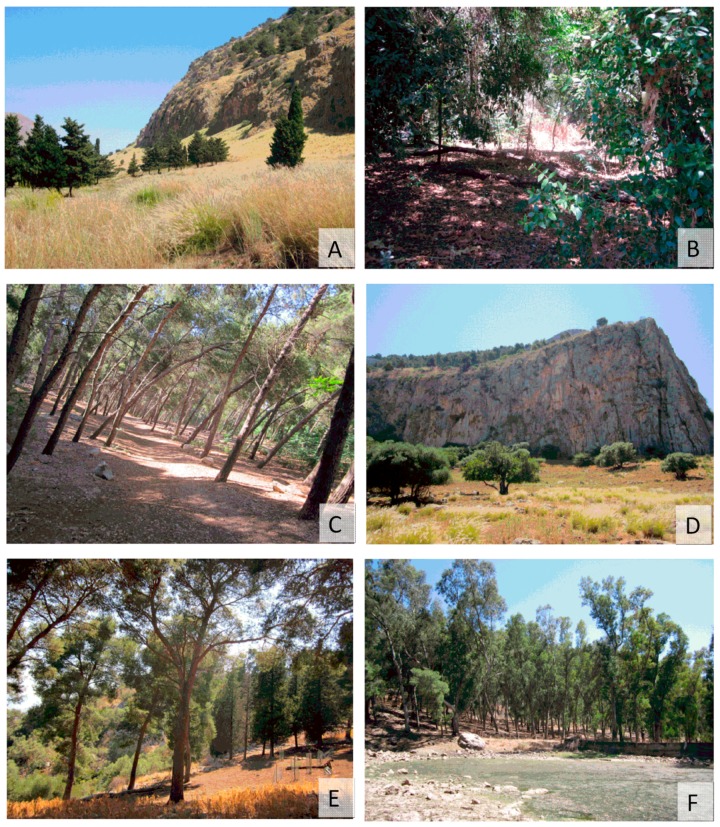
(**A**–**F**) Collection sites in the Natural Reserve of Monte Pellegrino monitored in this study. (**A**) Site n° 1. *Sede Landolina*; (**B**) Site n° 2. *Boschetto Airoldi*; (**C**) Site n° 3. *Pineta Ex Scuderie Reali*; (**D**) Site n° 4 *Sito Valdesi*; (**E**) Site n° 5. *Castello Utveggio*; and (**F**) Site n° 6. *Gorgo S. Rosalia.*

Monitoring was carried out in the Natural Reserve of Monte Pellegrino, a regional natural reserve established in 1996, situated in the northern part of Palermo (Sicily, Italy, Figure 1). Monte Pellegrino extends over an area of about 1300 hectares (ha), and it strongly characterizes the image of Palermo, bordering the city in the north and extending out over the Mediterranean Sea. Its expanse is 1050 ha and includes the whole mountain of Monte Pellegrino (Zone A—Reserve) and the Real Tenuta Favorita (Zone B—Pre-reserve). The Reserve, with its Mediterranean climate, hosts a rich fauna and flora; it is characterized by a diffuse artificial forest, but it also includes a wide variety of natural environments, with a considerable biodiversity and the presence of some endemic species [30,31,32]. The Reserve constitutes an important area for recreation activities of many inhabitants of Palermo. Six monitoring sites were selected (Figure 2A–F): Site n° 1. *Sede Landolina* (Lon 13.33809; Lat 38.17215; 76 m above sea level a.s.l.); Site n° 2. *Boschetto Airoldi* (Lon 13.35141; Lat 38.14946; 35 m a.s.l.); Site n° 3. *Pineta Ex Scuderie Reali* (Lon 13.34295; Lat 38.16512; 98 m a.s.l.); Site n° 4 *Sito Valdesi* (Lon 13.33418; Lat 38.18920; 20 m a.s.l.); Site n° 5. *Castello Utveggio* (Lon 13.35469; Lat 38.15640; 280 m a.s.l.) and Site n° 6. *Gorgo S. Rosalia* (Lon 13.35179; Lat 38.17005; 392 m a.s.l.). The sites were chosen taking into consideration differences in habitat, vegetation, and frequency of human activities. Site n° 1 was characterized by a sparse vegetation, shrubs, and herbaceous plants. Site n° 2 contained artificial forest (pine, eucalyptus) and it was characterized by the presence of rabbits (*Oryctolagus cuniculus*; Linnaeus, 1758), rodents (*Rattus norvegicus*; Berkenhout, 1769), and some species of *Canidae*. In site n° 3 natural forest with shadow areas were present, while site n° 4 was characterized by shrubs, herbaceous plants, and the presence of goats. The last two sites showed the presence of artificial forest with pine and cypress in site n° 5 and pine, eucalyptus, and a little lake in site n° 6.

Geographical coordinates of each site were recorded by Global Positioning System (GPS) with Roma1940 reference system, using East—North coordinate pairs.

### 2.2. Ticks Collection and Identification

Ticks were collected by dragging method every two weeks for two years from June 2012 to May 2014 and stored in 70% ethyl alcohol. Collected arthropods were identified according to morphological keys [33,34,35,36].

### 2.3. Ecological Analysis

Analysis of environmental characteristics of tick collection sites was carried out by processing and overlapping different information levels through Environmental Systems Research Institute (ESRI) ArcGIS 9.3 software [37]. Analysed levels were the digital elevation model (DEM), regional technical maps, land cover (Corine land cover), and forest vegetation.

### 2.4. Data Analysis

Data were processed taking into account the sampling month, collection site, and tick species in order to define how the density of the collected ticks varied in time (during the months of the year) and in space (in the different collection sites). Charts were drawn showing the percentage of the species identified at each site.

### 2.5. Processing of Maps with Proportionate Circles

Monthly maps related to the two years of monitoring were drawn using ESRI ArcGIS 9.3 (Redlands, CA, USA). Maps provide epidemiological information with circles, whose size is proportional to the number of ticks collected in the monitored sites.

## 3. Results

### 3.1. Ecological Analysis

**Figure 3:**
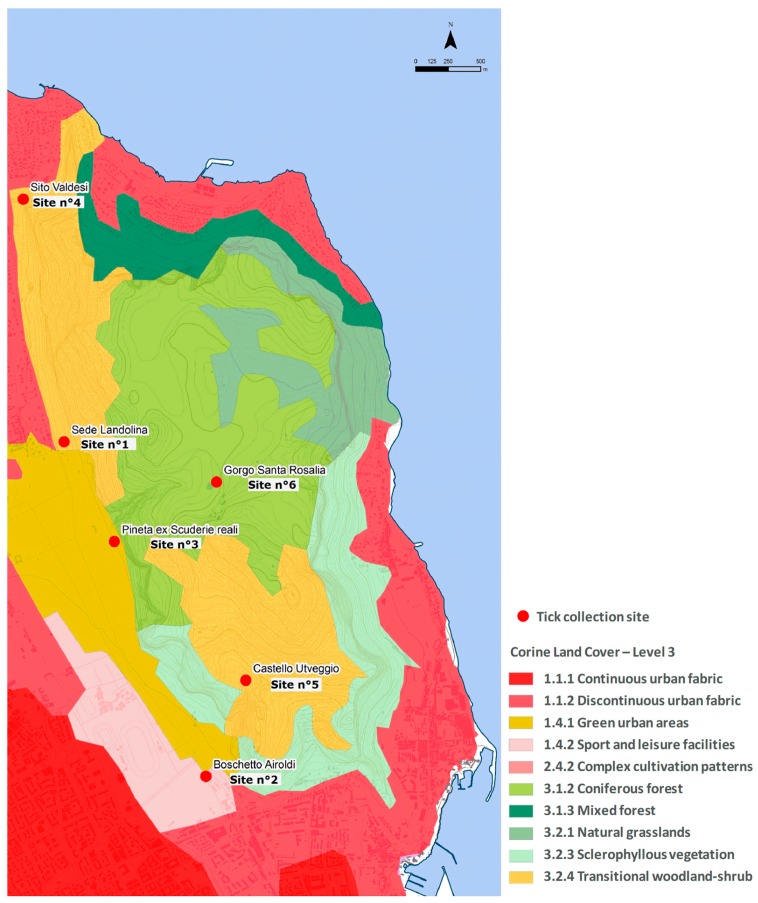
Corine Land Cover (2006). Land use.

**Figure 4:**
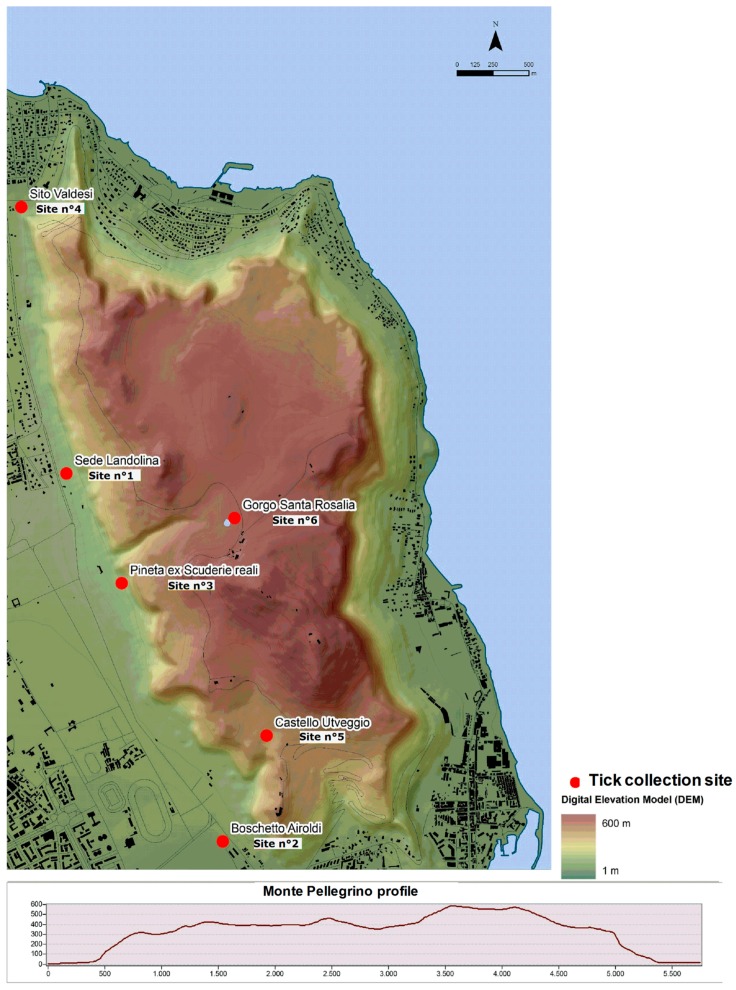
Digital Elevation Model (above) and Profile of Monte Pellegrino (below).

**Figure 5:**
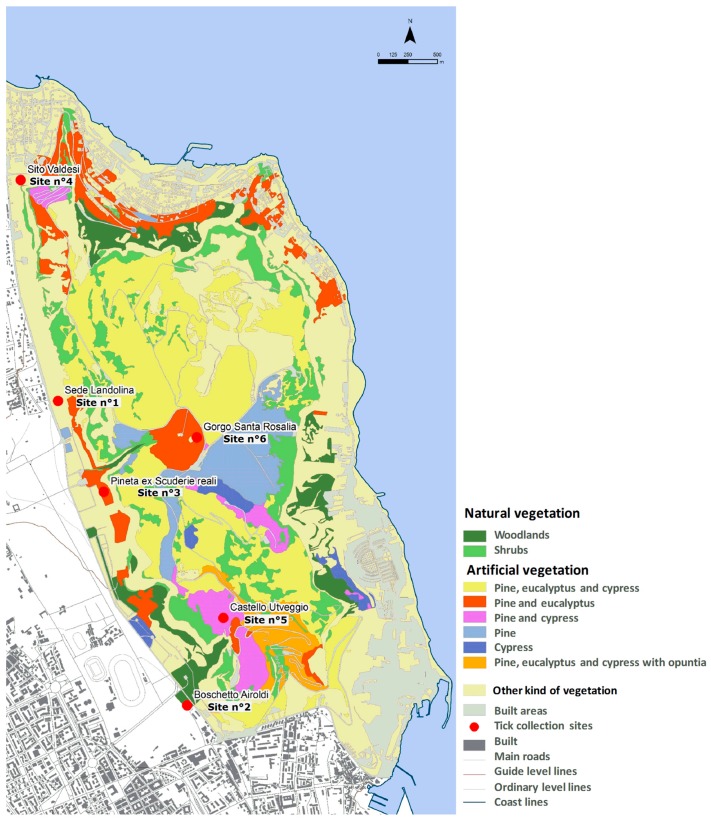
Forest vegetation map of Monte Pellegrino.

Environmental characteristics of tick collection sites were analysed according to the land use (Corine land cover), forest vegetation, and DEM, and the obtained maps are showed in Figure 3, Figure 4 and Figure 5, respectively. Corine land cover analysis showed that sites n° 1, 4, and 5 fall within the Transitional woodland-shrub area, with sites n° 1 and n° 4 at the border with Discontinuous Urban Fabric Area and, for site n° 1, also with a Green Urban Area. Site n° 2 is characterized by Sport/Leisure Facilities, site n° 3 is within a Green Urban Area, and site n° 6 entirely falls in a Coniferous Forest. In particular, the Forest Vegetation map of Monte Pellegrino showed that sampling sites fell into areas of natural vegetation (woodlands, site n° 2), artificial vegetation (pine and cypress/eucalyptus, site n° 3, n° 5, and n° 6), and other kind of vegetation (mainly herbaceous vegetation, sites n° 1 and n° 4).

Digital elevation model of Monte Pellegrino showed an altitude range of 1–600 m; sites n° 1 to n° 4 were located at low altitude (<100 m), whilst sites n° 5 and n° 6 were located at moderate altitudes (280–392 m a.s.l.).

### 3.2. Ticks Abundance

A total of 3092 ticks (1728 in the first year and 1364 in the second year) were collected, comprising seven different species: *Ixodes ventalloi* (Gil Collado, 1936) (46.09%), *Hyalomma lusitanicum* (Koch, 1844) (19.99%), *Rhipicephalus sanguineus* (17.34%), *Rhipicephalus pusillus* (Gil Collado, 1936) (16.11%), *Haemaphisalis sulcata* (0.36%), *Dermacentor marginatus* (0.10%), and *Rhipicephalus turanicus* (Pomerantsev, 1936) (0.03%). Most of the collected ticks were adults (number (n.) 2582, of which n. 1276 were males and n. 1306 females), n. 495 were nymphs and only n. 20 were larvae. Nymphs belonged to *I. ventalloi*, *H. lusitanicum,* and *R. sanguineus* species, whilst larvae belonged to *I. ventalloi* and *R. sanguineus* species.

#### 3.2.1. Monthly Trend

**Figure 6:**
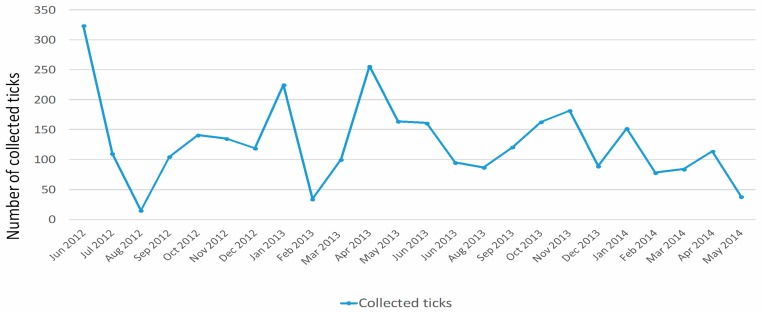
Monthly trend of collected ticks during the months of monitoring.

The monthly trend of collected ticks is shown in Figure 6. Highest tick numbers were found in June 2012 (n. 324), April 2013 (n. 256), and January 2013 (n. 225), while August 2012 (n. 15), February 2013 (n. 34), and May 2014 (n. 38) were the months with the lowest tick numbers. Table 1 shows for each tick species the number of specimens collected in the different months of the year. *I. ventalloi* showed a peak in January 2013 (n. 221), preceded by December 2012 (n. 117); furthermore, a considerable presence of such species was reported from October 2013 to January 2014 (max. n. 177, min. n. 89). *H. lusitanicum* was abundant during the first months of monitoring activity (June–July 2012, n. 181 and 94, respectively) and moderately present from June to October 2013. *R. sanguineus* had a peak in April 2013 (n. 159) that was followed by a moderate but constant presence until September 2013. *R. pusillus* was abundant in June 2012 (n. 67), May–June 2013 (n. 88 and n. 96), and April 2014 (n. 98). The other three species (*H. sulcata*, *D. marginatus*, and *R. turanicus*) were only occasionally found within the monitoring period.

#### 3.2.2. Spatial Distribution

**Figure 7:**
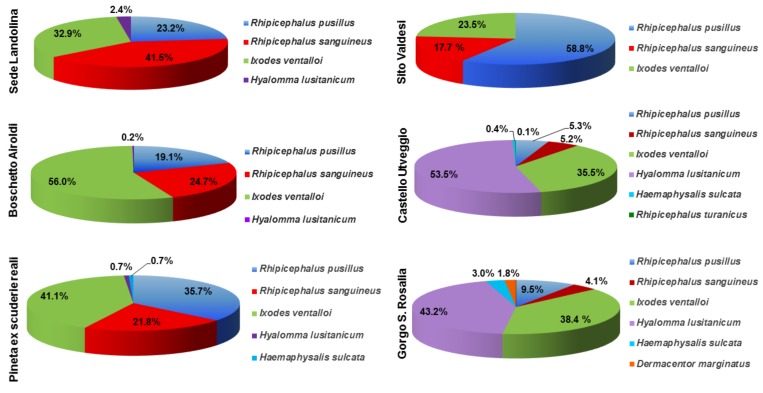
Percentage values of each tick species in the different collection sites.

Among the different collection sites, the highest numbers of ticks were collected in sites n° 2 (n. 1522 ticks) and n° 5 (n. 1005 ticks) and the lowest number in site n° 4 (n. 34 ticks), as reported in Table 2. Percentage values of each tick species in all the collection sites are reported in Figure 7.

Site n° 2 was mainly characterized by a high presence of *I. ventalloi* (n. 853 ticks) and a comparable number of *R. sanguineus* and *R. pusillus* (n. 376 and n. 290, respectively); differently, site n° 5 showed a high presence of *H. lusitanicum* (n. 538), followed by *I. ventalloi* (n. 357). Data from site n° 3 showed a moderate presence of *I. ventalloi*, *R. pusillus*, and *R. sanguineus* (n. 115, n. 100, and n. 61, respectively), while a similar number of *H. lusitanicum* (n. 73) and *I. ventalloi* (n. 65) were observed in site n° 6. Ticks from sites n° 1 (total number of ticks: 82) and n° 4 (total number of ticks: 34), where the lowest number of vectors were collected, mainly belonged to the species *R. pusillus*, *R. sanguineus*, and *I. ventalloi*.

**Figure 8:**
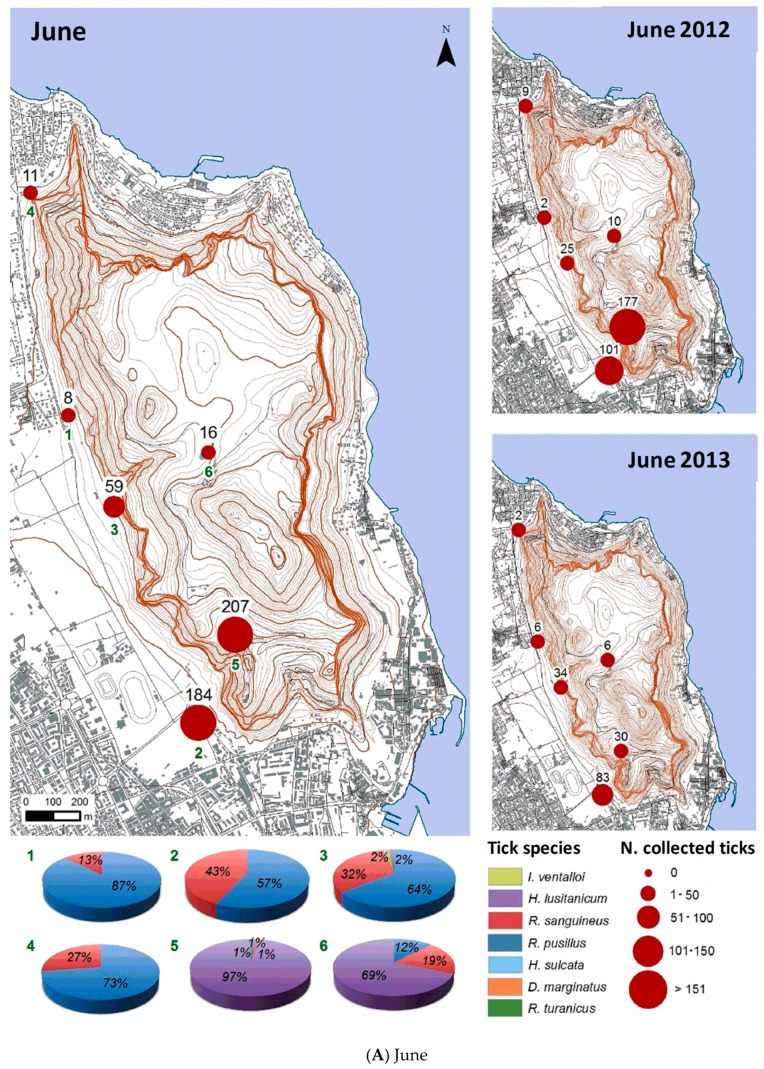

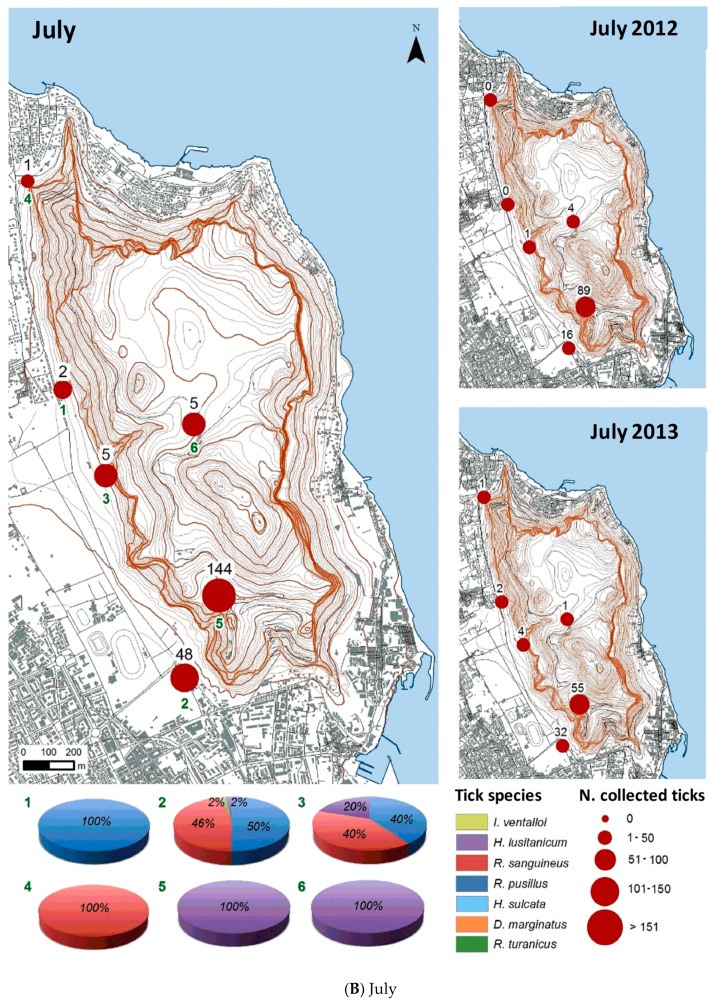

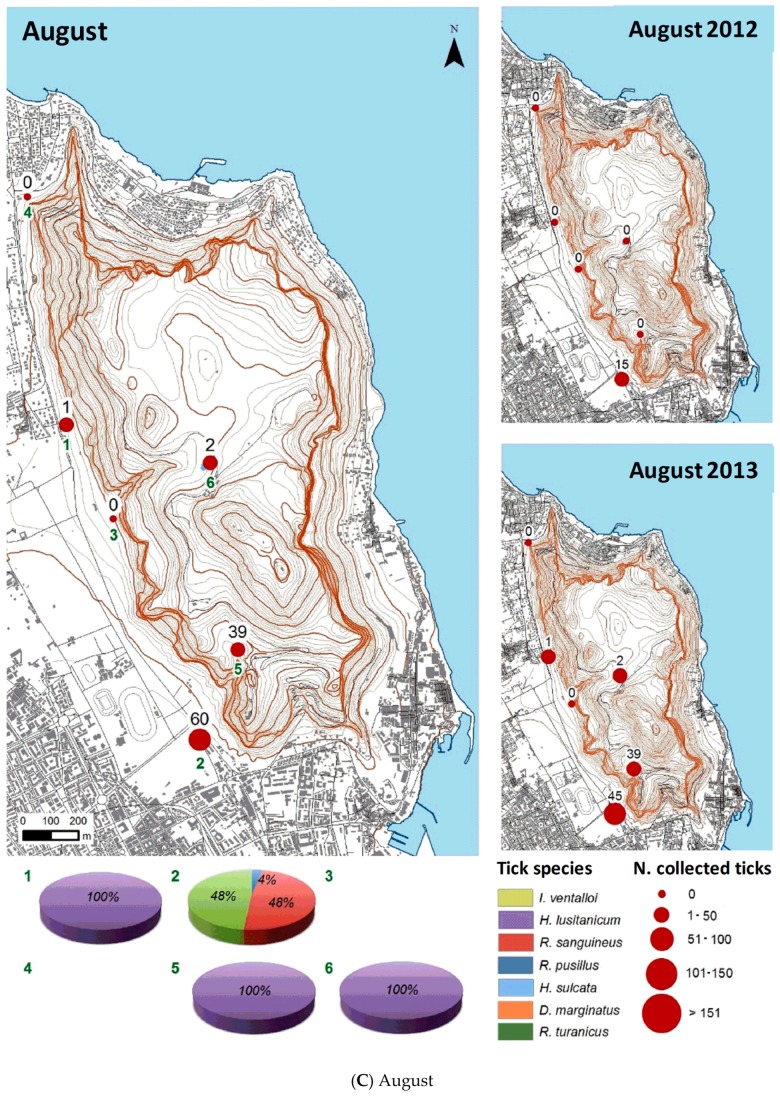

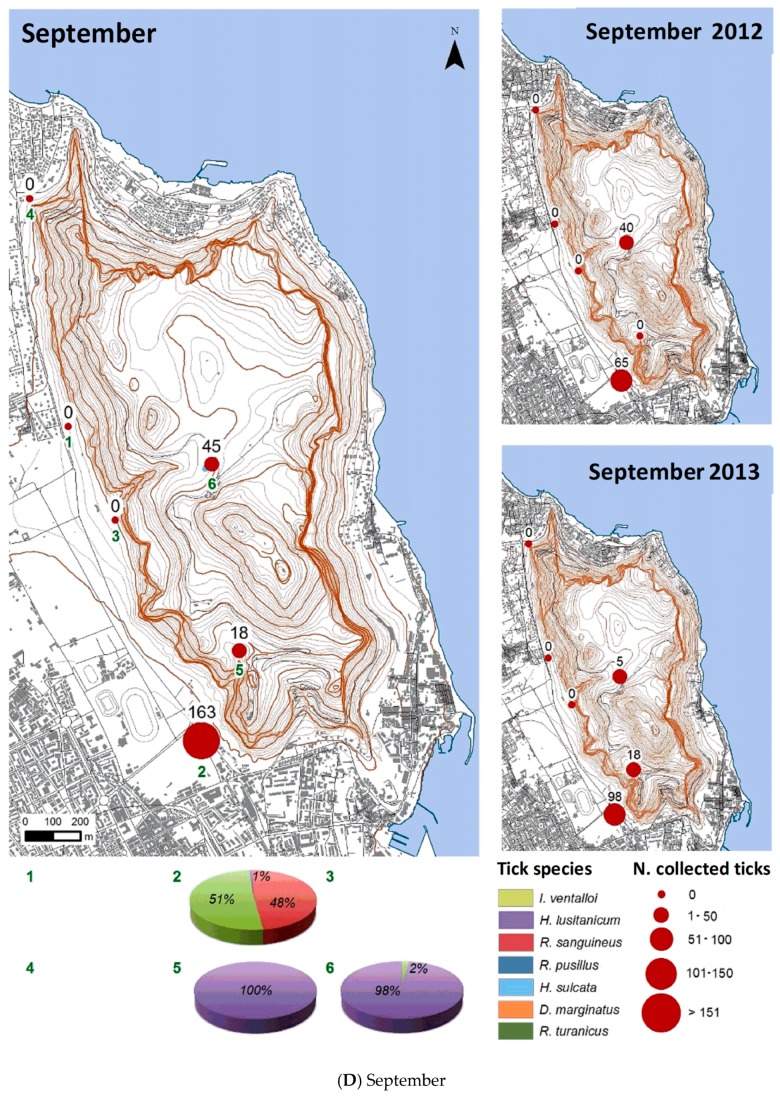

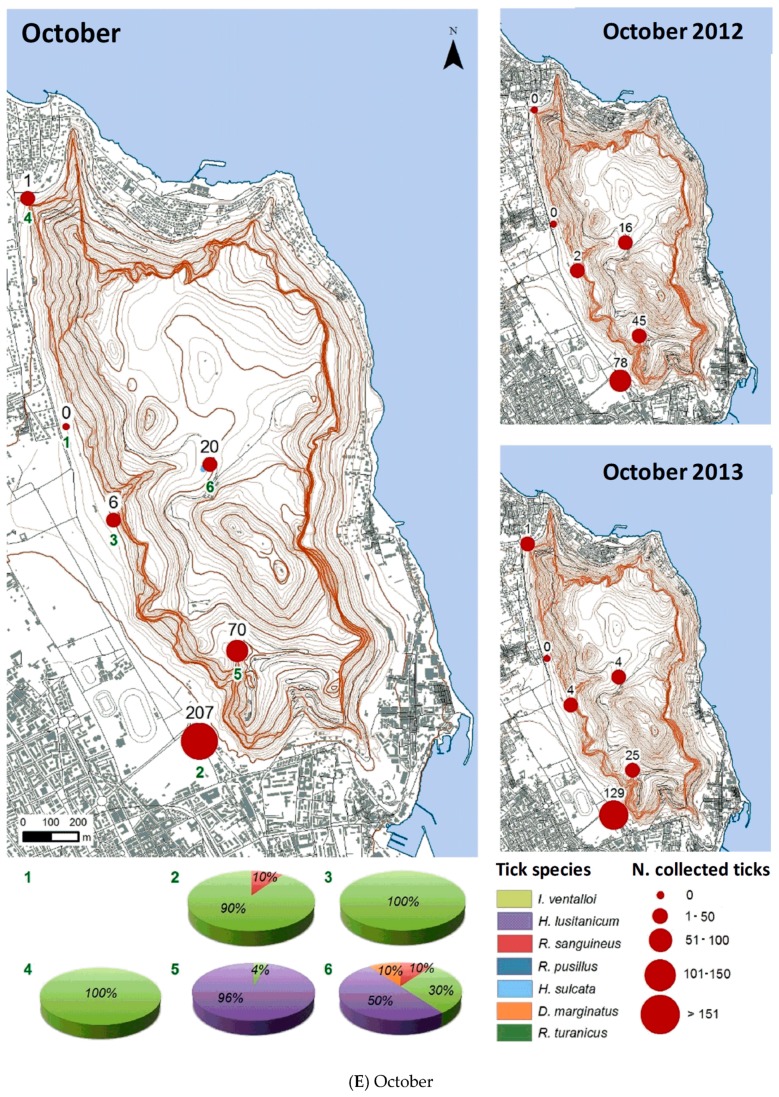

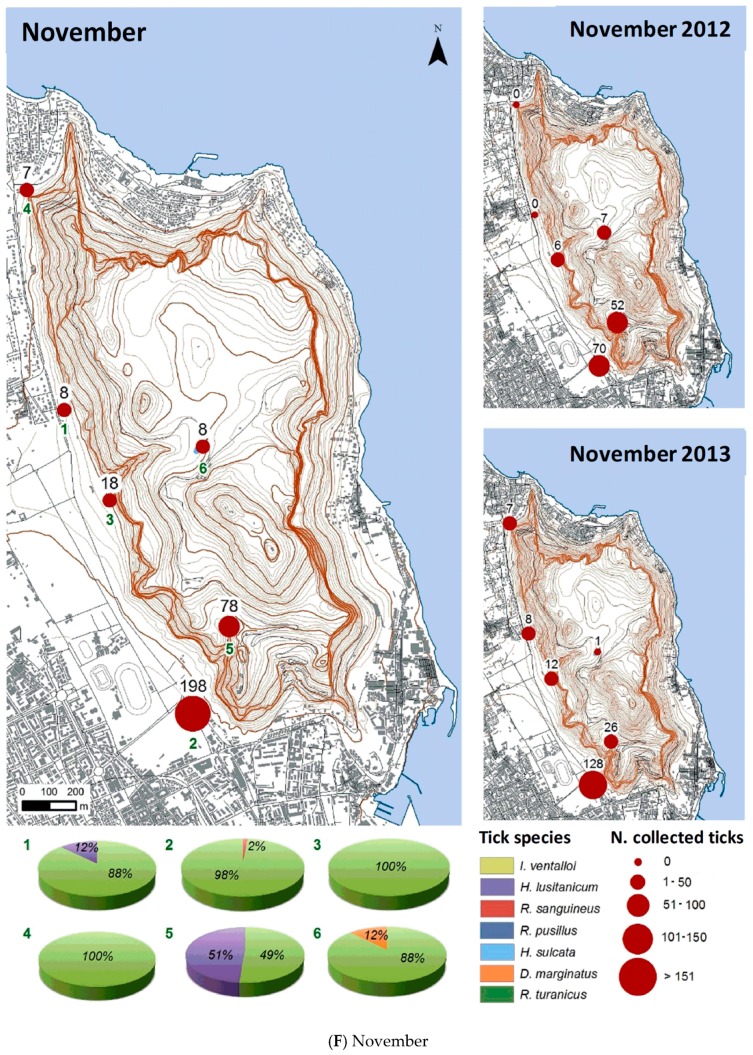

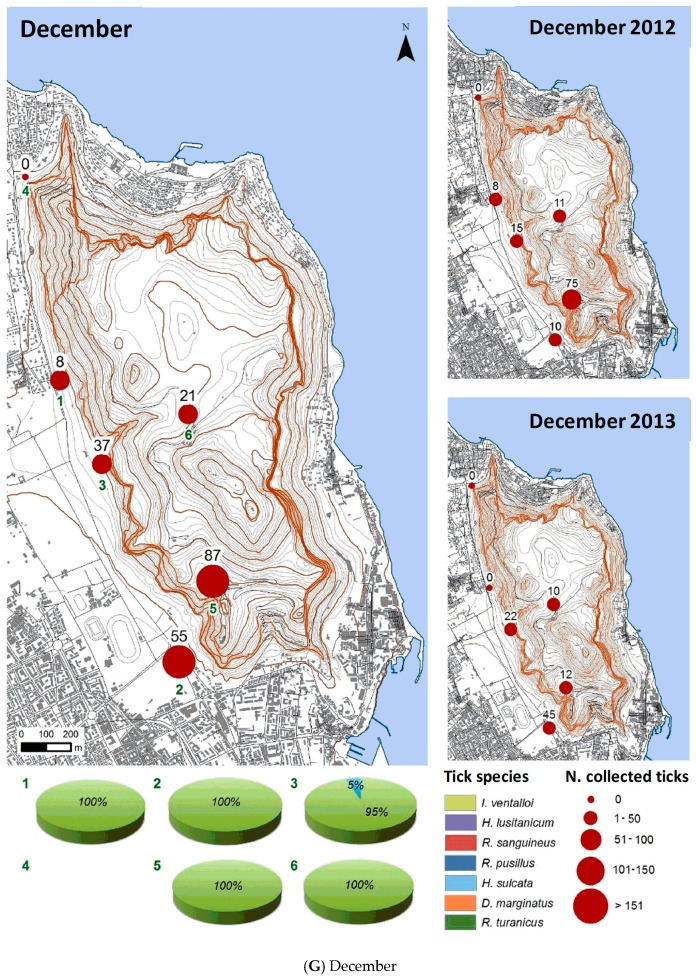

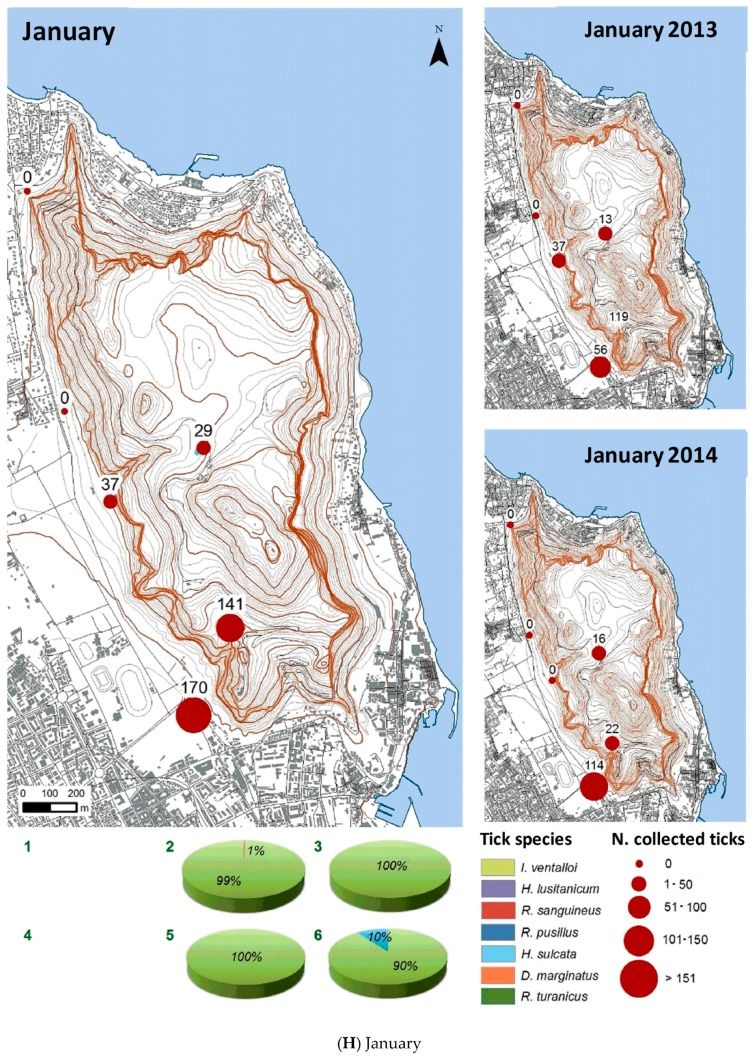

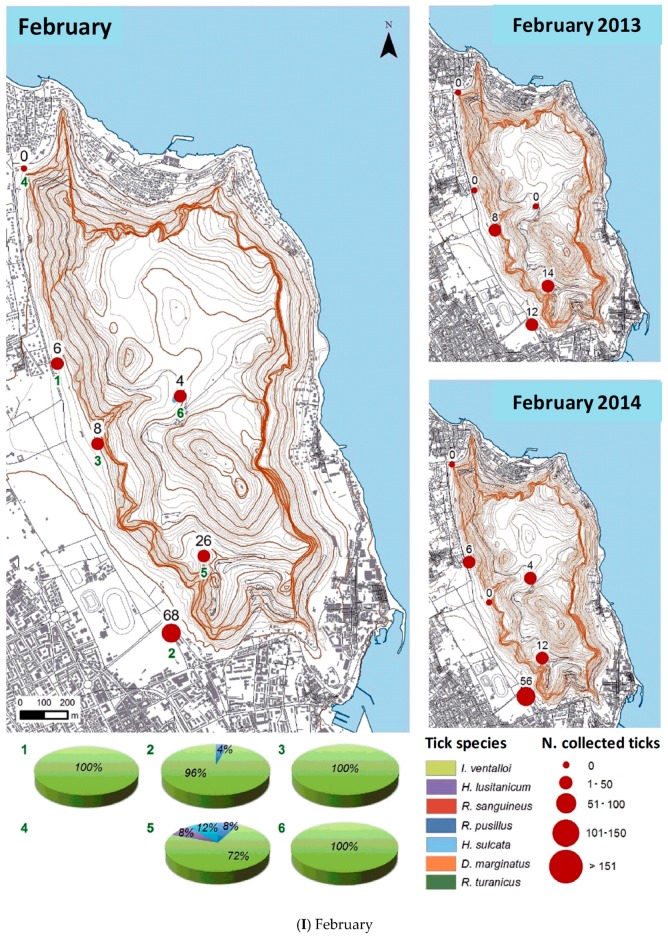

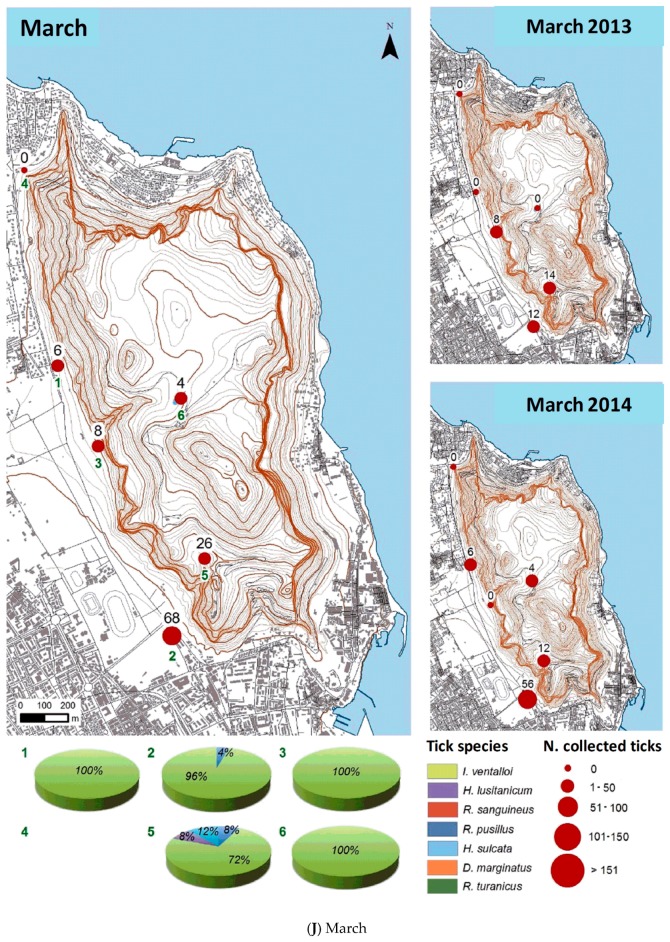

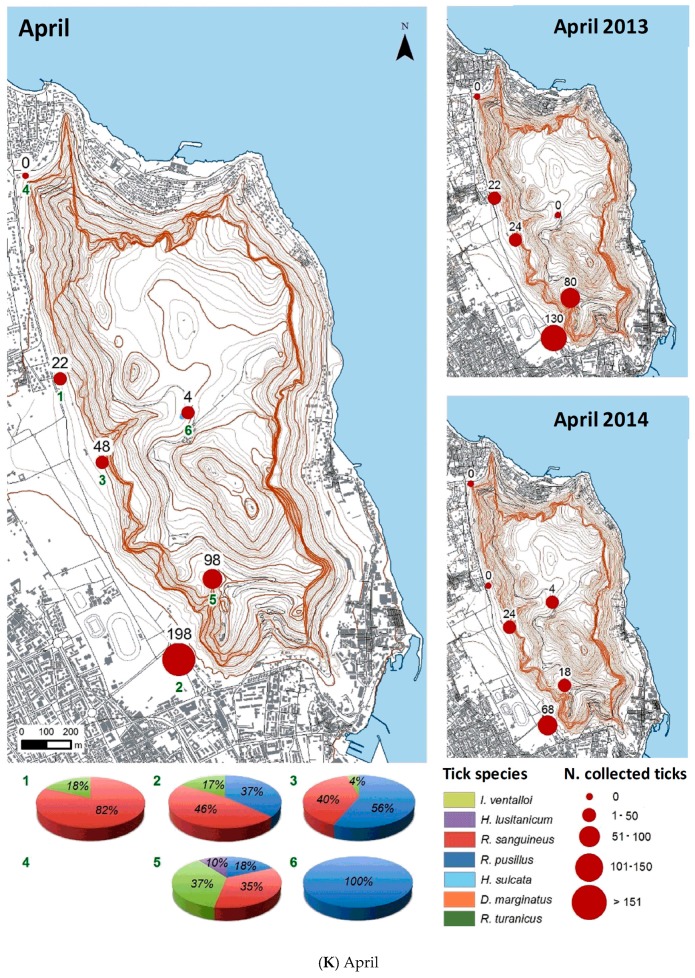

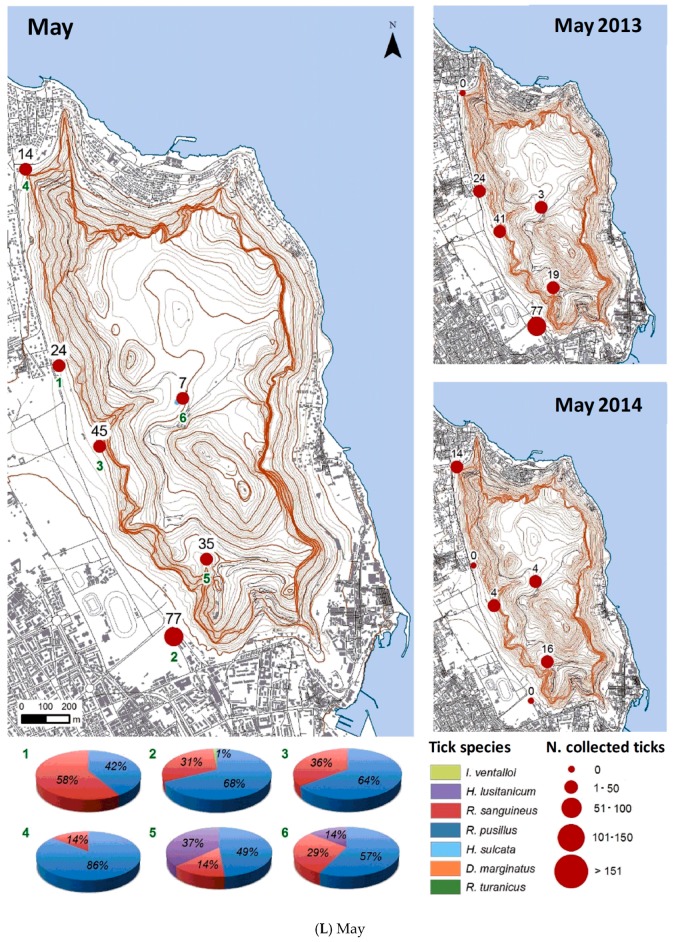
(**A**–**L**) Monthly maps related to the two years of monitoring from June (**A**) to May (**L**). Circle sizes in the maps are proportional to the number of ticks collected in the monitored sites. Each map reports not only a monthly value as a result of the two-year period, but also the values separately for each year of the study. At the bottom of each monthly map, charts showing tick species composition in each collection site are reported.

Monthly maps with circles proportional to the tick number were created using the geographical information systems (Figure 8A–L). In addition to the values for the two-year period, maps referring to each year of the study were created (Figure 8A–L).

## 4. Discussion

In recent years, increasing attention has been directed to ticks and tick-borne pathogens and particularly to zoonotic agents. Many factors have, indeed, led to an increased contact between people and these arthropods, as for example climate changes [38], the increase of wild animals in rural and peri-urban areas [39], and the increased interest of people in outside activities [22].

The analysis reported in this study describes the results of a two-year survey conducted to investigate the presence of ticks in Monte Pellegrino Natural Reserve of Palermo (Italy), a peri-urban park attended by citizens for sportive and recreational activities.

The study provides information on tick distribution among collection sites with different environmental characteristics during each month and in correlation to the environmental characteristics of the territory (altimetry, land cover, and vegetation).

A great diversity of tick species was found in the Natural Reserve. Data analysis showed that sites having similar environmental features (1, 2, and 3 and 5 and 6) were characterized by similar patterns of tick species. In fact, sites n° 1, 2, and 3 shared the presence of *I. ventalloi*, *R. pusillus*, and *R. sanguineus*, although site-specific differences were present. Site n° 1 showed a low and comparable presence of the three species; site n° 2 was characterized by a dominance of *I. ventalloi* followed by similar numbers of *R. pusillus* and *R. sanguineus*. Finally, *R. pusillus* and *I. ventalloi* were equally present in site n° 3, followed by *R. sanguineus*. Of particular interest is the high presence of *I. ventalloi* in site n° 2 that may be related to different factors, such as a favourable natural (woodland) vegetation, evidenced by field observations and GIS analysis, as well as the presence of wild rabbits and cats. In fact, these animals are reported as preferential hosts for such tick species, which have been proposed as potential vectors for various pathogens (*Rickettsia helvetica*, *Anaplasma phagocytophilum*, *Rickettsia monacensis*, and *Bartonella clarridgeiae* (Lawson and Collins 1996)) and Eyach virus (Rehse-Küpper et al., 1976) [40,41]. Thus, site n° 2 may represent an important location for potential pathogens transmission from *I. ventalloi*, and monitoring activity should be enhanced to determine the effective public health risk in this area.

Sites n° 5 and n° 6 showed the greatest richness of tick species, in particular of *H. lusitanicum* and *I. ventalloi*. The relevant presence of *H. lusitanicum* in site n° 5, characterized by moderate altitude and artificial vegetation (Pine and Cypress), may require further attention given the vector role of such species for pathogens such as *Theileria annulata* (Dschunkowsky and Luhs, 1904) [42].

Considering all the sites, the most abundant tick species were *I. ventalloi* (n. 1425), *H. lusitanicum* (n. 618), *R. sanguineus* (n. 536), and *R. pusillus* (n. 498). All these tick species are proven or suspected vectors of animal and human pathogens (Table 3). Indeed, as aforementioned, *I. ventalloi* is a potential vector for several zoonotic bacteria, i.e., *Rickettsia helvetica*, *Rickettsia monacensis*, *Anaplasma phagocytophilum*, and *Bartonella clarridgeiae* [41]. *R. sanguineus* is a tick species of veterinary and public health significance as it is vector of *Anaplasma marginale* (Theiler, 1910), *Babesia bigemina* (Smith and Kilborne, 1893), and *Babesia bovis* (V. Babes, 1888). The tick, also known as the brown dog tick, is a parasite to a number of hosts, including humans, and it is a relevant vector of important zoonotic agents, such as *Rickettsia* spp. [13]. *H. lusitanicum* tick species are a frequent parasite of large mammals such as cattle, sheep, goats, and pigs, and it is mainly associated with the transmission of *Anaplasma* spp. and *T. annulata* [42]. Trans-ovarian transmission of *Coxiella burnetii* has also been documented for this tick.

Months in which the highest number of total ticks were collected were June 2012 and April and January 2013, while the lowest numbers of ticks were collected in August 2012, February 2013, and May 2014. The presence and/or absence of ticks during the year changed according to the life cycle of the different species. The graphs joined to the monthly maps (Figure 8A–L) show the monthly trend of each identified tick species in each collection site, highlighting the differences in seasonality of each species due to its own peculiar biological cycle.

In fact, *I. ventalloi* was present mostly from autumn to spring, with the greatest abundance between October and January. On the contrary, *H. lusitanicum* was collected mainly from late spring to autumn. *R. sanguineus* and *R. pusillus* showed a similar seasonal trend, and their number was higher in spring and summer, while significantly decreasing to zero in winter. The few specimens of the ticks belonging to the other identified species (*H. sulcata*, *D. marginatus*, and *R. turanicus*) did not allow establishing the period of abundance/scarcity of these species.

## 5. Conclusions

To our knowledge, the area analysed in this study was not previously subjected to any entomological investigation concerning ticks. This study contributes to the understanding and mapping of the presence and distribution of ticks and it can lead to a powerful surveillance integrated strategy. These data also allow for the identification of periods of greater tick abundance in the study area, thus providing indications for precautions to be observed by the attenders during such periods.

The study constitutes a premise for additional research, including any correlation among pathogens in ticks, microclimate, and hosts distribution analysis. Various studies in northern and central Italy [22,54,55] analysed the presence of pathogens in ticks collected from urban parks, identifying different agents of zoonoses. In our study, almost all tick species found were already reported as potential or recognized vectors of a wide range of animal/human pathogens (i.e., *Rickettsia* spp., *A. phagocytophilum*, *Coxiella burnetii*, *Bartonella clarridgeia*, *Babesia* spp., and *Theileria* spp.), reinforcing the need for control programs within the area aiming to reduce both tick abundance and the impact of environmental factors favouring tick development and spread. The GIS-based ecological analysis allowed for the analysis of information on specific suitable habitats for such tick species in the area, providing a useful base for control interventions.

The comprehensive depiction of ticks and TBPs in the area would represent a useful tool for decision support for Health Authorities to define possible risk for specific TBDs and, thus, to choose adequate strategies of pest control for the preservation of public human health.

## Figures and Tables

**Table 1:** Number of collected ticks for each month of monitoring, reported for each tick species.

Month	Total Tick Number	*Ixodes ventalloi*	*Hyalomma lusitanicum*	*Rhipicephalus sanguineus*	*Rhipicephalus pusillus*	*Haemaphysalis sulcata*	*Dermacentor marginatus*	*Rhipicephalus turanicus*
June 2012	324	0	181	76	67	0	0	0
July 2012	110	0	94	10	6	0	0	0
August 2012	15	6	0	9	0	0	0	0
September 2012	105	39	39	27	0	0	0	0
October 2012	141	69	52	18	0	0	2	0
November 2012	135	97	34	3	0	0	1	0
December 2012	119	117	0	0	0	2	0	0
January 2013	225	221	0	1	0	3	0	0
February 2013	34	32	0	0	1	1	0	0
March 2013	100	32	2	35	28	3	0	0
April 2013	256	65	8	159	24	0	0	0
May 2013	164	1	14	61	88	0	0	0
June 2013	161	1	32	31	96	0	0	1
July 2013	95	1	57	15	22	0	0	0
August 2013	87	23	42	20	2	0	0	0
September 2013	121	45	25	51	0	0	0	0
October 2013	163	134	25	4	0	0	0	0
November 2013	182	177	5	0	0	0	0	0
December 2013	89	89	0	0	0	0	0	0
January 2014	152	152	0	0	0	0	0	0
February 2014	78	70	2	0	4	2	0	0
March 2014	84	44	4	10	26	0	0	0
April 2014	114	10	2	4	98	0	0	0
May 2014	38	0	0	2	36	0	0	0
Total	3092	1425	618	536	498	11	3	1

**Table 2:** Number of collected ticks for each collection site, reported for each tick species.

Collection Site	Total	*R. pusillus*	*R. sanguineus*	*I. ventalloi*	*H. lusitanicum*	*H. sulcata*	*D. marginatus*	*R. turanicus*
*Sede Landolina*	82	19	34	27	2	0	0	0
*Boschetto Airoldi*	1522	290	376	853	3	0	0	0
*Pineta Ex Scuderie Reali*	280	100	61	115	2	2	0	0
*Sito Valdesi*	34	20	6	8	0	0	0	0
*Castello Utveggio*	1005	53	52	357	538	4	0	1
*Gorgo S. Rosalia*	169	16	7	65	73	5	3	0
	3092	498	536	1425	618	11	3	1

**Table 3:** Principal habitat for each tick species found in this study with reference to associated pathogens/human diseases.

Tick Species	Principal Habitat (GIS Forest Vegetation Map)	Pathogens Associated	Human Diseases	References
*I. ventalloi*	Natural vegetation (Woodland)	*R. helvetica* * R. monacensis * A. phagocytophilum * Bartonella clarridgeia * Eyach virus *	SFGR HGA CSD Eyach Virus Disease	[41,43]
*H. lusitanicum*	Artificial vegetation (Pine and Cypress)	CCHFV * (Simpson et al., 1967) [44] *Coxiella burnetii* * R. aeschlimannii * Theileria annulata *Babesia* spp.	CCHF SFGR Q fever	[13,42,45,46]
*R. sanguineus*	Natural vegetation (Woodland)	*R. conorii* * R. rickettsii * (Brumpt, 1922) *Coxiella burnetii* * Babesia canis (Piana & Galli-Valerio, 1895) *Ehrlichia canis* (Donatien and Lestoquard 1935) *Anaplasma marginale*	MSF RMSF Q fever	[47,48,49]
*R. pusillus*	Natural vegetation (Woodland)	*R. sibirica* mongolitimonae * (Fournier et al., 2006) [50] Coxiella burnetiid *	Lymphangitis-associated rickettsiosis Q fever	[51,52]
*Hae. sulcata*	Artificial vegetation (Pine and cypress/eucalyptus)	*Anaplasma ovis* (Lestoquard, 1924) *Theileria annulata*	-	[33]
*D. marginatus*	Artificial vegetation (Pine and eucalyptus)	*R. sibirica* * (Zdrodovskii, 1948) *R. slovaca* * R. aeschlimannii * Babesia canis	Siberian tick typhus SFGR SENLAT	[53]
*R. turanicus*	Artificial vegetation (Pine and cypress)	*R. monacensis* * R. massiliae * R. conorii * R. aeschlimannii * Babesia spp.	MSF SFGR	[54]

GIS: Geographical Information System; CCHF: Crimean-Congo Hemorrhagic Fever; CSD: Cat-Scratch Disease; HGA: Human granulocytic anaplasmosis; MSF: Mediterranean Spotted Fever; RMSF: Rocky Mountain Spotted Fever; SENLAT: Scalp eschar and Neck Lymph Adenopathy; SFGR: Spotted Fever Group Rickettsioses. * Asterisks indicate human relevant pathogens.
